# Bard Versus the 2022 American Society of Plastic Surgeons In-Service Examination: Performance on the Examination in Its Intern Year

**DOI:** 10.1093/asjof/ojad066

**Published:** 2023-07-19

**Authors:** Daniel Najafali, Erik Reiche, Sthefano Araya, Justin M Camacho, Farrah C Liu, Thomas Johnstone, Sameer A Patel, Shane D Morrison, Amir H Dorafshar, Paige M Fox

## Abstract

**Background:**

Bard is a conversational generative artificial intelligence (AI) platform released by Google (Mountain View, CA) to the public in May 2023.

**Objectives:**

This study investigates the performance of Bard on the American Society of Plastic Surgeons (ASPS) In-Service Examination to compare it to residents' performance nationally. We hypothesized that Bard would perform best on the comprehensive and core surgical principles portions of the examination.

**Methods:**

Google's 2023 Bard was used to answer questions from the 2022 ASPS In-Service Examination. Each question was asked as written with the stem and multiple-choice options. The 2022 ASPS Norm Table was utilized to compare Bard's performance to that of subgroups of plastic surgery residents.

**Results:**

A total of 231 questions were included. Bard answered 143 questions correctly corresponding to an accuracy of 62%. The highest-performing section was the comprehensive portion (73%). When compared with integrated residents nationally, Bard scored in the 74th percentile for post-graduate year (PGY)-1, 34th percentile for PGY-2, 20th percentile for PGY-3, 8th percentile for PGY-4, 1st percentile for PGY-5, and 2nd percentile for PGY-6.

**Conclusions:**

Bard outperformed more than half of the first-year integrated residents (74th percentile). Its best sections were the comprehensive and core surgical principle portions of the examination. Further analysis of the chatbot's incorrect questions might help improve the overall quality of the examination's questions.

The use of artificial intelligence (AI) and machine-learning (ML) applications has seen considerable growth in the medical literature.^1,2^ OpenAI (San Francisco, CA) has released a series of advanced open-access generative pretrained transformer (GPT) architectures, otherwise known as chatbots.^3^ These chatbots, such as ChatGPT and GPT-4, can comprehend a variety of inputs and provide outputs to users interacting with it in seconds (eg, answers to questions, generation of ideas, etc). ChatGPT was used by Kung et al to assess its performance on the United States Medical Licensing Examination, a multi-phased examination for medical licensing across all 3 levels (eg, Steps 1-3) and it passed all of them.^4^ On May 10, 2023, Google released its chatbot called Bard (Google; Mountain View, CA) to the public. Chatbots have been used for a variety of applications in plastic surgery.^5-11^

The American Society of Plastic Surgeons (ASPS) In-Service Examination^12^ is a standardized examination that residents take annually to evaluate their knowledge and prepare them for written and oral board examinations. The In-Service Examination is 250 total questions and lasts 5h and 55 min in duration. This examination has been noted to be difficult to study given that the best resources are dense and yet to be determined. The ASPS Education Network (ASPS EdNet) is a commonly used and centralized resource that plastic surgery programs engage with for the In-Service Examination. GPT-3.5, ChatGPT, has taken the In-Service Examination and performed well compared to first-year integrated residents.^13,14^

In theory, if chatbots are capable of meeting or exceeding the standards held for physicians on gateway examinations by performing at a satisfactory level or by demonstrating a superior knowledge base, then chatbots may have the potential to contribute to medical decision-making in a supportive fashion. The In-Service Examination is standardized and compiled with experts in the field of plastic surgery deliberating on the most relevant questions to assess a trainee's knowledge, thereby making it a valuable test case for the chatbot. Here, we explore Google's Bard's performance on the 2022 ASPS In-Service Examination to determine its accuracy on plastic surgery questions. We hypothesized that Bard would be able to successfully generate answers to most questions and perform highest on the comprehensive and core surgical principles portions of the examination. Bard was evaluated and summarized via performance metrics based on its answers.

## METHODS

### Natural Language Processing Artificial Intelligence (AI)

Google's Bard was the large language model utilized in this study. Bard was released to the public on May 10, 2023.

### 2022 ASPS In-Service Examination, ASPS Educational Network, and Bard Input Formatting

Each question stem was asked as written, with the accompanying multiple-choice options. These questions were given directly to Bard. If any error or inability to provide an answer was encountered due to the question relating to medical diagnosis or advice, thus prompting the chatbot to return a disclaimer that it cannot answer the question (eg, “I am not a medical professional…”), Bard was prompted 3 more times maximum until an answer was given. If no answer was given, the question was deemed indeterminate and removed from the final analysis. Question prompts with graphics or images in addition to text that contained stems or information were included. All questions that the examination committee subsequently removed prior to scoring due to possible content ambiguity or poor statistical performance were excluded from the analysis, as reported in Supplemental Table 1. Any questions that solely required analyzing a graphic or image to correctly answer the questions were excluded due to limitations with inputting figures into the chatbot. Each examination question was also assigned the corresponding sections: (1) Section 1: Comprehensive, (2) Section 2: Hand and Lower Extremity, (3) Section 3: Craniomaxillofacial, (4) Section 4: Breast and Cosmetic, and (5) Section 5: Core Surgical Principles.

Using the ASPS EdNet, listed categories (major) and subcategories (minor) were assigned to each question. Supplemental Table 2 lists each of the ASPS EdNet designations that could correspond to each question type. Each corresponding categorization was tabulated by a plastic surgery resident, and 3 independent reviewers assessed the tabulation of those categories.

### Bard Performance Evaluation and Grading of Responses for Accuracy

The 2022 ASPS Norm Table was utilized to compare Bard's performance on the examination to that of subgroups of plastic surgery residents to compare its abilities to those of residents historically. This provided a final percentile that could be used to determine Bard's overall score based on its percent correct.

### Statistical Analysis

All data were entered into a standardized Microsoft Excel spreadsheet (Microsoft Corporation, Redmond, WA). Descriptive statistics were presented with frequency (percentage; eg, *N* [%]). Categorical variables were compared using a χ^2^ test with Yates correction or Fisher's exact test, as appropriate. Statistical analyses were performed with R (version 4.1.0) and RStudio (version 1.4.1717) software (Boston, MA). All 2-sided *P*-values <.05 were considered statistically significant (eg, *P* < .05).

## RESULTS

A total of 231 questions from the original 250 were included in the final analysis. The performance metrics for each subsection of the examination are provided in Figure. Accuracy was 62% overall (Table 1). A total of 6 questions were indeterminate. With respect to indeterminate responses, specifically those omitted because Bard could not provide an answer to them, if they were censored/included Bard's accuracy for the examination would be 63%/60%, respectively. The comprehensive portion of the examination had the highest accuracy (72.9%), followed by the core surgical principles section (72.1%). The worst performing section in terms of accuracy was the breast and cosmetic (52.3%). ASPS EdNet major and minor categories are presented in Supplemental Table 3. Performance in EdNet major categories is summarized in Table 2. The fundamentals of surgery had the highest accuracy (73.7%), with breast surgery having the lowest accuracy (40.9%).

**Figure.**
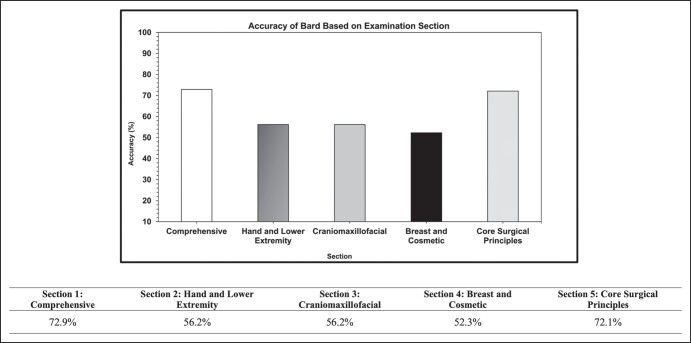
Performance of Bard (Google) on the 2022 Plastic Surgery In-Service Examination stratified by section. Six questions were indeterminate and were not included in the final accuracy calculation.

**Table 1.** Norm Table Corresponding to the Accuracy Achieved by Google's Bard (Mountain View, CA) Compared to OpenAI's GPT-3.5 Platform (San Francisco, CA)

Chatbot	Total 2022 test % correct	Independent program				Integrated program					
		All	First year	Second year	Third year	First year	Second year	Third year	Fourth year	Fifth year	Sixth year
Bard	62%	25	56	23	16	74	34	20	8	1	2
Humar et al GPT-3.5	57%	12	24	7	10	49	13	5	4	0	0

GPT, generative pretrained transformer.

**Table 2.** EdNet Major Categories and Accuracy Metrics for Bard (Google; Mountain View, CA)

Major category	Aesthetic	Breast	Fundamentals of surgery	Gender affirmation surgery	Head and neck	Lower extremity	Nonclinical	Trunk	Upper extremity
Accuracy, *n*/*N* Bard	12/22	9/22	42/57	1/2	36/63	5/8	10/13	9/13	19/31
Accuracy, % Bard	54.5	40.9	73.7	50.0	57.1	62.5	76.9	69.2	61.3

EdNet, educational network.

Based on the 2022 ASPS Norm Table and overall accuracy, Bard performed at the 74th percentile compared to first-year residents from integrated programs. A national comparison across resident years is given for trainees from both the integrated and independent pathways for all studies investigating the accuracy of chatbots on the 2022 In-Service Examination (Table 1).

## DISCUSSION

In this study, we thoroughly assessed Bard's performance on the 2022 ASPS In-Service Examination. The answers that Bard provided were graded against the answer key provided by the ASPS. When evaluating its performance using the 2022 ASPS Norm Tables, it scored in the 74th percentile compared to integrated interns. Bard performed best on the comprehensive and core surgical principles sections.

### Bard's Performance on the 2022 In-Service Examination by Section

The Plastic Surgery In-Service Training Examination has been noted to be difficult to study given that the best resources are dense and yet to be determined. Frojo et al conducted a literature review on evidence-based essentials for preparing for the Plastic Surgery In-Service Examination and found that preparation should include a review of landmark articles and current publications in select plastic surgery journals supplemented by core textbooks.^15^ The chatbot scored best in the comprehensive category (∼73%), followed by core surgical principles (∼72%), hand and lower extremity (56%) and craniomaxillofacial (56%), and breast and cosmetic (52%). This is likely because there are more widely available resources for the classical topics of the comprehensive and core surgical principles sections that the chatbot was exposed to in its training data. Meaike et al highlight that the core surgical principles portion of the In-Service Examination has the most variety and encompasses a wide variety of literature, making it difficult to prepare for in comparison to other sections.^16^ This may also indicate that the information is more widely available and covers a more expansive range of resources applicable to other domains. Moreover, these sections may be more generalizable to other specialties of surgery which may account for Bard's higher performance and reflect of its baseline knowledge. It is also possible that the questions in the comprehensive section were more direct or written in a fashion that the chatbot better understood. The authors hypothesized that performance would be higher in the comprehensive and core surgical principal sections of the In-Service Examination primarily due to the fact that the questions focus on testing a resident's ability to logically reason. Logical thinking and conversational exchanges are the foundation of many large language models and their training structures. Large language models are heavily based on the principles of probability; thus, it inherently makes sense that they could draw on and respond to principles that are foundational to the surgical specialty. Furthermore, the breadth of data the models have been trained on and currently have access to allows them to answer very complex and comprehensive questions but may not allow them to think in an abstract manner that other sections may require the tester to utilize in addition to their foundational knowledge. While the resources are incredible in large language models, it does not take away the subtleties learned in actual practice, which are appropriately applied by the user during the examination.

Additionally, the chatbot has a limitation given its experimental status, which may have prevented it from sourcing data related to plastic surgery–specific questions based on recent articles. In the plastic surgery literature, Google's Bard has been used, in part, for a commentary to demonstrate its ability to answer questions relevant to breast augmentation.^17^ While ChatGPT has received significant attention in the field of academic research, Bard has received very little and has not had its potential explored in plastic surgery regarding its possible contributions. We would be remiss not to acknowledge that this is also a driver of our study.

Bard demonstrated superior performance when compared with OpenAI's ChatGPT for the 2022 Plastic Surgery In-Service Examination. Although it may seem as though it outperformed ChatGPT at a degree that is superficial or marginal in nature, even when we include the questions that we excluded and assume they are incorrect, which was done in accordance with Kung et al's study that was the landmark paper for chatbots taking a standardized medical examination, its accuracy is still 60% overall. This places its performance in the 64th percentile for first-year integrated residents. When compared with the previously reported accuracy of 57% (placing it in the 49th percentile for first-year integrated residents), this is substantial; however, we report an overall accuracy of 62%, which corresponds to the 74th percentile for first-year integrated residents. What may seem like a small difference in accuracy, surmounts to a large delta when comparing the performance to the ASPS Norm Tables—which is in alignment with what programs do with their residents' scores. Chatbot performance has many implications. Historically, physicians have had to dedicate significant time to research and literature review to ensure their practices are up-to-date with the most current evidence-based methods. This is why resources like UpToDate have been designed in the first place, which aim to eliminate that burden. Therefore, it is clear that large language models like Bard and ChatGPT warrant research in how we can further reduce physician fatigue and enhance our decision-making support tools.

The EdNet categories from ASPS were tabulated, as the ASPS EdNet is a go-to resource for resident education. This strategy of assigning 2022 In-Service Examination questions to EdNet major and minor categories gave more granularity to the topics that the chatbot was prompted on. This highlights areas that Bard performed better in or may have knowledge gaps in relating to plastic surgery. ASPS EdNet categories with the most coverage or highest proportion of questions on the 2022 In-Service Examination were fundamentals of surgery and head and neck, with an accuracy of 73.7% and 57.1%, respectively. Whereas a topic that encompassed a lower proportion of 2022 In-Service Examination questions was gender affirmation surgery, which had <50% accuracy on 2 questions. Given that gender affirmation surgery and the lower extremity topics from ASPS EdNet were given a much lower emphasis on the examination compared to other areas, it will be important to conduct a more thorough review of the subspecialty areas. A recent study using OpenAI's ChatGPT to answer 2576 questions pertaining to otolaryngology subspecialties demonstrates that the chatbot requires further refinement as it has a higher propensity to get certain questions incorrect depending on the topic area.^18^

### Analysis of Bard's Incorrect Questions

The chatbot got 38% of questions (*n* = 88) incorrect. Given that it has been found that performance on the Plastic Surgery In-Service Examination can predict success in the American Board of Plastic Surgery Examination, the ASPS is motivated to improve the quality and objectivity of this examination.^19^ A possible future direction of large language models is that they can be used to generate question banks or can be utilized to possibly improve the writing of questions. Since these models can be trained with plugins or with data from incorrect answers, we believe that this could be a potential area that warrants future investigation. Chatbots identifying questions that may require improvement, aim to enhance the overall quality of the In-Service Examination. By analyzing these questions, one can pinpoint areas where enhancements could be made, such as improving clarity and writing techniques, ensuring the availability of adequate data to support the correct answer, providing access to supporting articles that are behind paywalls, and ensuring equal access to data for all residents. Using the chatbot’s feedback could facilitate the creation of a more effective examination.

When prompted regarding its training, Bard responds that it cannot be directly trained by users but can learn from its interactions with them. Bard also currently provides drafts which are alternative answers that users can go through and select if they do not find the initial response appropriate. These drafts can also be regenerated by the user. The chatbot notes that it is still under development. The large language model is certainly limited in its knowledge and could also be thinking of certain questions in a fashion that residents are trained not to. However, it will always be a possibility that the chatbot is just getting the answer incorrect, because it lacks the information needed to answer the question.

### Limitations

This is an early and experimental release of Bard, which means that with additional iterations, training data, may expand and performance may improve. Bard tends to generate answers that appear to be well developed with sources, but it does not provide references that can be double-checked in each case. There could also be errors inherent in prompting, and the ideal input method may not have been captured by the technique explored in this study. To mitigate this, we used a similar methodology to Humar et al.^13^ There are certainly limitations inherent to Bard. It sometimes stated: “I’m unable to help you with that, as I’m only a language model and don’t have the necessary information or abilities.” The chatbot would often require prompting multiple times and even had a cap on the number of queries that could be executed at 1 time, similar to other chatbots by OpenAI. At the time of this study, the chatbot was unable to answer questions that contained images or graphics, which limited its capacity to answer certain questions. Perhaps future iterations of Google's Bard will be capable of doing tasks that include user images. No questions from previous In-Service Examinations were given to the chatbot. Thus, no answers from prompts before the 2022 examination were explored, and it is unclear if this would have impacted the overall performance of the chatbot.

## CONCLUSIONS

This study examined Bard's performance on the 2022 ASPS In-Service Examination. Bard's accuracy placed it in the 74th percentile compared to first-year integrated plastic surgery residents. This is an early release of the AI software. As updates and additional chatbot platforms are introduced, applications will grow. Plastic surgeons should explore their capabilities to develop use cases. Just as surgical trainees quickly learn and adapt to their new environment, we are excited to see how chatbots adapt and learn as time goes on.

## Supplementary Material

ojad066_Supplementary_Data
