# Supplementary material for: Bard Versus the 2022 American Society of Plastic Surgeons In-Service Examination: Performance on the Examination in Its Intern Year
Source: Aesthet Surg J Open Forum. 2023 Jul 19;6:ojad066. doi: 10.1093/asjof/ojad066 (PMC10776237; doi:10.1093/asjof/ojad066)
Supplement: ojad066_Supplementary_Data [file ojad066_supplementary_data.zip › 23-0059_Supplemental Table 1_with ref.docx]

**Supplemental Table 1**: Included Questions in the Final Analysis From the 2022 Plastic Surgery In-Service Examination

| Section | Questions in Section |
| --- | --- |
| Comprehensive | 48 |
| Hand and Lower Extremity | 48 |
| Craniomaxillofacial | 48 |
| Breast and Cosmetic | 44 |
| Core Surgical Principles | 43 |
| Total: 231^*^ | |

^*^Per the 2022 ASPS In-Service Self-Assessment Examination for Residents Computation and Interpretation of Test Scores Norm Tables: “The 2022 Educational Assessment Examination consisted of 250 total test items. However, item 7 from the Comprehensive subtest, item 113 from the Craniomaxillofacial subtest, items 169, 183, and 188 from the Breast and Cosmetic subtest, and items 207 and 225 the Core Surgical Principles subtest were deleted prior to scoring because of possible content ambiguity or poor statistical performance. Please make note of these deleted items in your syllabus. Participants were awarded one point for each correct response on total test items. The maximum total test raw score of 243 points…”^12^

The remainder of questions were removed because of difficulty prompting them with the chatbot: 93 (diagram of the carpal tunnel contents), 150 (image of ear anomaly), 176 (image of ear deformity), 233 (formatting has multiple columns that need to be considered in answer choices), 234 (sonographic image), 246 (ultrasound image)

Six questions were unable to be answered by Bard: 2, 55, 153, 187, 218, 238.
