# Supplementary material for: Bard Versus the 2022 American Society of Plastic Surgeons In-Service Examination: Performance on the Examination in Its Intern Year
Source: Aesthet Surg J Open Forum. 2023 Jul 19;6:ojad066. doi: 10.1093/asjof/ojad066 (PMC10776237; doi:10.1093/asjof/ojad066)
Supplement: ojad066_Supplementary_Data [file ojad066_supplementary_data.zip › 23-0059_Supplemental Table 2.docx]

**Supplemental Table 2**. EdNet Major and Minor Categories

| ASPS EdNet Major Categories | | | | | | | | |
| --- | --- | --- | --- | --- | --- | --- | --- | --- |
| Fundamentals of Surgery | Head and Neck | UE | Breast | Gender Affirmation Surgery | Trunk | LE | Aesthetic | Non-Clinical |
| ASPS EdNet Minor Categories | | | | | | | | |
| Anesthesia | Cleft Lip | Patient Safety | Anatomy | Gender Affirmation Surgery | Abdominal Wall Reconstruction | Foot Reconstruction | Blepharoplasty | Evolution of Evidence and Biostatistics |
| Burns | Cleft Lip Nose | Arthritis | Congenital Breast Anomalies |  | Anatomy, Physiology, Embryology | Muscle Flaps of the LE | Body Contouring | Evidence-Based Medicine |
| Critical care | Cleft palate | Congenital hand | Breast augmentation |  | Chest wall reconstruction | Fasciocutaneous flaps of the le | Brow lift | Icd-10 and cpt coding |
| Benign skin lesions | Congenital head and neck anomalies | Dupuytren's disease | Gynecomastia |  | Congenital disorders | Reconstruction of vascular lesions | Face and neck lift | Interpersonal communication |
| Flaps and Grafts | Craniofacial Anomalies | Extensor Tendon | Breast Cancer |  | Pelvic Reconstruction | Targeted Muscle Reinnervation of the LE | Hair Restoration | Leadership |
| Geriatrics and End of Life | Facial Fractures | Flexor Tendon | Breast Reconstruction (Ancillary Based) |  | Pressure Sores | Traumatic Injuries | Lasers | Medical Photography |
| Implants and Biomaterials | Head and Neck Cancer | Fractures and Dislocations | Breast Reconstruction (Autogenous Based) |  | Spinal Reconstruction | Vascular Surgery | Liposuction | Medicolegal |
| Necrotizing infections | Microtia and ear reconstruction | Hand infections | Breast reconstruction (implant based) |  |  | Venous insufficiencies of the le | Migraine surgery | Outpatient operating facility |
| Malignant skin lesions | Nasal reconstruction | Hand tumors | Breast reduction and hypertrophy |  |  |  | Non-surgical rejuvenation | Patient safety |
| Microsurgery | Orthognathic Surgery | Ligamentous Injuries | Ptosis and Mastopexy |  |  |  | Oculoplastic Surgery | Payment Systems |
| Neurosurgery | Scalp, Forehead, and Skull Reconstruction | Nerve Compression |  |  |  |  | Rhinoplasty | Physician Wellness |
| Orthopedic surgery | Soft-tissue injuries of the face | Nerve injury |  |  |  |  | Skin care | Professionalism and ethics |
| Pediatric surgery | Vascular anomalies | Replantation revascularization |  |  |  |  |  | Business practice risk management |
| Pharmacology and Therapeutics | Velopharyngeal Insufficiency | Soft Tissue Coverage of the UE |  |  |  |  |  | The Business of Medical Practice |
| Special Techniques, Minimally Invasive, and Non-Invasive Techniques | Facial Nerve Injury | Vascular Trauma and Disease of the Hand |  |  |  |  |  |  |
| Thoracic surgery | Facial nerve injury | Wrist injuries |  |  |  |  |  |  |
| Transplantation and Immunology |  | Targeted Muscle Reinnervation of the UE |  |  |  |  |  |  |
| Trauma |  |  |  |  |  |  |  |  |
| Wound healing |  |  |  |  |  |  |  |  |

ASPS, American Society of Plastic Surgeons; CPT, Current Procedural Terminology; EdNet, educational network; ICD-10, International Classification of Diseases, Tenth Revision; UE, upper extremity; LE, lower extremity.
