# Supplementary material for: Bard Versus the 2022 American Society of Plastic Surgeons In-Service Examination: Performance on the Examination in Its Intern Year
Source: Aesthet Surg J Open Forum. 2023 Jul 19;6:ojad066. doi: 10.1093/asjof/ojad066 (PMC10776237; doi:10.1093/asjof/ojad066)
Supplement: ojad066_Supplementary_Data [file ojad066_supplementary_data.zip › 23-0059_Supplemental Table 3.docx]

**Supplemental Table 3.** EdNet Categories Evaluated in the 2022 Plastic Surgery In-Service Examination

| EdNet Category | Level | Section 1: Comprehensive | Section 2: Hand and Lower Extremity | Section 3: Craniomaxillofacial | Section 4: Breast and Cosmetic | Section 5: Core Surgical Principles | *P* |
| --- | --- | --- | --- | --- | --- | --- | --- |
| EdNet Major Category N, (%) | Aesthetic | 2 (4.2) | 0 (0.0) | 1 (2.1) | 19 (43.2) | 0 (0.0) | <0.001 |
|  | Breast | 5 (10.4) | 0 (0.0) | 0 (0.0) | 13 (29.5) | 4 (9.3) |  |
|  | Fundamentals of Surgery | 22 (45.8) | 5 (10.4) | 2 (4.2) | 5 (11.4) | 23 (53.5) |  |
|  | Gender Affirmation Surgery | 0 (0.0) | 0 (0.0) | 2 (4.2) | 0 (0.0) | 0 (0.0) |  |
|  | Head and Neck | 7 (14.6) | 8 (16.7) | 43 (89.6) | 2 (4.5) | 3 (7.0) |  |
|  | Lower Extremity | 3 (6.2) | 5 (10.4) | 0 (0.0) | 0 (0.0) | 0 (0.0) |  |
|  | Non-Clinical | 0 (0.0) | 0 (0.0) | 0 (0.0) | 4 (9.1) | 9 (20.9) |  |
|  | Trunk | 9 (18.8) | 1 (2.1) | 0 (0.0) | 1 (2.3) | 2 (4.7) |  |
|  | Upper Extremity | 0 (0.0) | 29 (60.4) | 0 (0.0) | 0 (0.0) | 2 (4.7) |  |
| EdNet Minor Category N, (%) | Abdominal Wall Reconstruction | 1 (2.1) | 0 (0.0) | 0 (0.0) | 0 (0.0) | 0 (0.0) | <0.001 |
|  | Aesthetic | 1 (2.1) | 0 (0.0) | 1 (2.1) | 2 (4.5) | 0 (0.0) |  |
|  | Anatomy | 0 (0.0) | 0 (0.0) | 0 (0.0) | 3 (6.8) | 0 (0.0) |  |
|  | Anatomy, Physiology, Embryology | 3 (6.2) | 0 (0.0) | 0 (0.0) | 1 (2.3) | 1 (2.3) |  |
|  | Anesthesia | 0 (0.0) | 0 (0.0) | 0 (0.0) | 2 (4.5) | 3 (7.0) |  |
|  | Arthritis | 0 (0.0) | 2 (4.2) | 0 (0.0) | 0 (0.0) | 0 (0.0) |  |
|  | Benign Skin Lesions | 2 (4.2) | 0 (0.0) | 0 (0.0) | 0 (0.0) | 1 (2.3) |  |
|  | Blepharoplasty | 0 (0.0) | 0 (0.0) | 0 (0.0) | 2 (4.5) | 0 (0.0) |  |
|  | Body Contouring | 1 (2.1) | 0 (0.0) | 0 (0.0) | 2 (4.5) | 0 (0.0) |  |
|  | Breast Augmentation | 0 (0.0) | 0 (0.0) | 0 (0.0) | 1 (2.3) | 0 (0.0) |  |
|  | Breast Cancer | 1 (2.1) | 0 (0.0) | 0 (0.0) | 2 (4.5) | 3 (7.0) |  |
|  | Breast Reconstruction (Autogenous Based) | 3 (6.2) | 0 (0.0) | 0 (0.0) | 0 (0.0) | 0 (0.0) |  |
|  | Breast Reconstruction (Implant Based) | 1 (2.1) | 0 (0.0) | 0 (0.0) | 4 (9.1) | 1 (2.3) |  |
|  | Burns | 0 (0.0) | 1 (2.1) | 0 (0.0) | 0 (0.0) | 2 (4.7) |  |
|  | Chest Wall Reconstruction | 2 (4.2) | 0 (0.0) | 0 (0.0) | 0 (0.0) | 0 (0.0) |  |
|  | Cleft Lip | 0 (0.0) | 0 (0.0) | 1 (2.1) | 0 (0.0) | 0 (0.0) |  |
|  | Cleft Palate | 0 (0.0) | 0 (0.0) | 2 (4.2) | 0 (0.0) | 0 (0.0) |  |
|  | Congenital Breast Anomalies | 2 (4.2) | 0 (0.0) | 0 (0.0) | 2 (4.5) | 1 (2.3) |  |
|  | Congenital Disorders | 1 (2.1) | 0 (0.0) | 0 (0.0) | 0 (0.0) | 0 (0.0) |  |
|  | Congenital Hand | 0 (0.0) | 7 (14.6) | 0 (0.0) | 0 (0.0) | 0 (0.0) |  |
|  | Congenital Head and Neck Anomalies | 0 (0.0) | 0 (0.0) | 4 (8.3) | 0 (0.0) | 0 (0.0) |  |
|  | Craniofacial Anomalies | 0 (0.0) | 0 (0.0) | 5 (10.4) | 0 (0.0) | 0 (0.0) |  |
|  | Critical Care | 0 (0.0) | 0 (0.0) | 0 (0.0) | 0 (0.0) | 2 (4.7) |  |
|  | Dupuytren's Disease | 0 (0.0) | 1 (2.1) | 0 (0.0) | 0 (0.0) | 0 (0.0) |  |
|  | Evidence-Based Medicine | 0 (0.0) | 0 (0.0) | 0 (0.0) | 0 (0.0) | 1 (2.3) |  |
|  | Extensor Tendon | 0 (0.0) | 3 (6.2) | 0 (0.0) | 0 (0.0) | 1 (2.3) |  |
|  | Face and Neck Lift | 0 (0.0) | 0 (0.0) | 0 (0.0) | 3 (6.8) | 0 (0.0) |  |
|  | Facial Fractures | 0 (0.0) | 0 (0.0) | 4 (8.3) | 0 (0.0) | 0 (0.0) |  |
|  | Fasciocutaneous Flaps of the LE | 0 (0.0) | 1 (2.1) | 0 (0.0) | 0 (0.0) | 0 (0.0) |  |
|  | Flaps and Grafts | 2 (4.2) | 1 (2.1) | 1 (2.1) | 0 (0.0) | 0 (0.0) |  |
|  | Flexor Tendon | 0 (0.0) | 7 (14.6) | 0 (0.0) | 0 (0.0) | 0 (0.0) |  |
|  | Fractures and Dislocations | 0 (0.0) | 2 (4.2) | 0 (0.0) | 0 (0.0) | 1 (2.3) |  |
|  | Gender Affirmation Surgery | 0 (0.0) | 0 (0.0) | 2 (4.2) | 0 (0.0) | 0 (0.0) |  |
|  | Gynecomastia | 0 (0.0) | 0 (0.0) | 0 (0.0) | 2 (4.5) | 0 (0.0) |  |
|  | Hair Restoration | 0 (0.0) | 0 (0.0) | 0 (0.0) | 1 (2.3) | 0 (0.0) |  |
|  | Hand Tumors | 0 (0.0) | 3 (6.2) | 0 (0.0) | 0 (0.0) | 0 (0.0) |  |
|  | Head and Neck Cancer | 1 (2.1) | 0 (0.0) | 10 (20.8) | 0 (0.0) | 0 (0.0) |  |
|  | ICD-10 and CPT Coding | 0 (0.0) | 0 (0.0) | 0 (0.0) | 0 (0.0) | 2 (4.7) |  |
|  | Implants and Biomaterials | 2 (4.2) | 0 (0.0) | 0 (0.0) | 0 (0.0) | 0 (0.0) |  |
|  | Lasers | 0 (0.0) | 0 (0.0) | 0 (0.0) | 3 (6.8) | 0 (0.0) |  |
|  | Leadership | 0 (0.0) | 0 (0.0) | 0 (0.0) | 0 (0.0) | 1 (2.3) |  |
|  | Liposuction | 5 (10.4) | 1 (2.1) | 1 (2.1) | 0 (0.0) | 3 (7.0) |  |
|  | Malignant Skin Lesions | 3 (6.2) | 0 (0.0) | 0 (0.0) | 0 (0.0) | 0 (0.0) |  |
|  | Microsurgery | 0 (0.0) | 0 (0.0) | 3 (6.2) | 0 (0.0) | 0 (0.0) |  |
|  | Microtia and Ear Reconstruction | 0 (0.0) | 1 (2.1) | 0 (0.0) | 0 (0.0) | 0 (0.0) |  |
|  | Muscle Flaps of the LE | 1 (2.1) | 0 (0.0) | 2 (4.2) | 0 (0.0) | 0 (0.0) |  |
|  | Nasal Reconstruction | 0 (0.0) | 2 (4.2) | 0 (0.0) | 0 (0.0) | 0 (0.0) |  |
|  | Nerve Compression | 1 (2.1) | 4 (8.3) | 0 (0.0) | 0 (0.0) | 1 (2.3) |  |
|  | Nerve Injury | 0 (0.0) | 0 (0.0) | 0 (0.0) | 2 (4.5) | 0 (0.0) |  |
|  | Oculoplastic Surgery | 0 (0.0) | 0 (0.0) | 6 (12.5) | 0 (0.0) | 0 (0.0) |  |
|  | Orthognathic Surgery | 2 (4.2) | 0 (0.0) | 0 (0.0) | 0 (0.0) | 0 (0.0) |  |
|  | Orthopedic Surgery | 0 (0.0) | 0 (0.0) | 0 (0.0) | 1 (2.3) | 5 (11.6) |  |
|  | Patient Safety | 1 (2.1) | 0 (0.0) | 0 (0.0) | 0 (0.0) | 0 (0.0) |  |
|  | Pelvic Reconstruction | 4 (8.3) | 0 (0.0) | 0 (0.0) | 3 (6.8) | 5 (11.6) |  |
|  | Pharmacology and Therapeutics | 1 (2.1) | 1 (2.1) | 0 (0.0) | 0 (0.0) | 1 (2.3) |  |
|  | Pressure Sores | 0 (0.0) | 0 (0.0) | 0 (0.0) | 3 (6.8) | 0 (0.0) |  |
|  | Professionalism and Ethics | 0 (0.0) | 0 (0.0) | 0 (0.0) | 1 (2.3) | 0 (0.0) |  |
|  | Ptosis and Mastopexy | 1 (2.1) | 0 (0.0) | 0 (0.0) | 0 (0.0) | 0 (0.0) |  |
|  | Replantation Revascularization | 0 (0.0) | 0 (0.0) | 0 (0.0) | 4 (9.1) | 0 (0.0) |  |
|  | Rhinoplasty | 0 (0.0) | 0 (0.0) | 1 (2.1) | 0 (0.0) | 0 (0.0) |  |
|  | Scalp, Forehead, and Skull Reconstruction | 1 (2.1) | 1 (2.1) | 0 (0.0) | 0 (0.0) | 0 (0.0) |  |
|  | Soft Tissue Coverage of the UE | 0 (0.0) | 0 (0.0) | 4 (8.3) | 0 (0.0) | 1 (2.3) |  |
|  | Soft-Tissue Injuries of the Face | 0 (0.0) | 0 (0.0) | 0 (0.0) | 0 (0.0) | 1 (2.3) |  |
|  | Targeted Muscle Reinnervation of the LE | 0 (0.0) | 0 (0.0) | 0 (0.0) | 0 (0.0) | 1 (2.3) |  |
|  | Transplantation and Immunology | 1 (2.1) | 2 (4.2) | 0 (0.0) | 0 (0.0) | 0 (0.0) |  |
|  | Trauma | 0 (0.0) | 0 (0.0) | 1 (2.1) | 0 (0.0) | 0 (0.0) |  |
|  | Traumatic Injuries | 0 (0.0) | 1 (2.1) | 0 (0.0) | 0 (0.0) | 0 (0.0) |  |
|  | Vascular Anomalies | 0 (0.0) | 3 (6.2) | 0 (0.0) | 0 (0.0) | 0 (0.0) |  |
|  | Vascular Surgery | 2 (4.2) | 0 (0.0) | 0 (0.0) | 0 (0.0) | 0 (0.0) |  |
|  | Vascular Trauma and Disease of the Hand | 2 (4.2) | 2 (4.2) | 0 (0.0) | 0 (0.0) | 5 (11.6) |  |
|  | Venous Insufficiencies of the LE | 0 (0.0) | 1 (2.1) | 0 (0.0) | 0 (0.0) | 0 (0.0) |  |
|  | Wound Healing | 0 (0.0) | 1 (2.1) | 0 (0.0) | 0 (0.0) | 0 (0.0) |  |
|  | Wrist Injuries | 1 (2.1) | 0 (0.0) | 0 (0.0) | 0 (0.0) | 0 (0.0) |  |
|  | Wrist Ligamentous Injuries | 1 (2.1) | 0 (0.0) | 1 (2.1) | 2 (4.5) | 0 (0.0) |  |

,American Society of Plastic Surgeons; CPT, current procedural terminology; EdNet, educational network; ICD-10, International Classification of Diseases, Tenth Revision; LE, lower extremity; UE, upper extremity
